# Regulatory role of PI16 in autoimmune arthritis and intestinal inflammation: implications for Treg cell differentiation and function

**DOI:** 10.1186/s12967-024-05082-1

**Published:** 2024-04-02

**Authors:** Yuankai Sun, Shiyu Lin, Hui Wang, Lei Wang, Yulu Qiu, Feifei Zhang, Nannan Hao, Fang Wang, Wenfeng Tan

**Affiliations:** 1 Department of Rheumatology, The First Affiliated Hospital of Nanjing Medical University, Nanjing, 210029 China; 2 Department of Cardiology, The First Affiliated Hospital of Nanjing Medical University, Nanjing, 210029 China

**Keywords:** PI16, Treg, AIA, DSS-induced colitis, Treg/Th17 balance, Autoimmune disease

## Abstract

**Background:**

Regulatory T cells (Tregs) are crucial in maintaining immune homeostasis and preventing autoimmunity and inflammation. A proportion of Treg cells can lose Foxp3 expression and become unstable under inflammation conditions. The precise mechanisms underlying this phenomenon remain unclear.

**Methods:**

The PI16 gene knockout mice (PI16^fl/fl^Foxp3^Cre^) in Treg were constructed, and the genotypes were identified. The proportion and phenotypic differences of immune cells in 8-week-old mice were detected by cell counter and flow cytometry. Two groups of mouse Naïve CD4^+^T cells were induced to differentiate into iTreg cells to observe the effect of PI16 on the differentiation and proliferation of iTreg cells, CD4^+^CD25^+^Treg and CD4^+^CD25^−^ effector T cells (Teff) were selected and co-cultured with antigen presenting cells (APC) to observe the effect of PI16 on the inhibitory ability of Treg cells in vitro. The effects of directed knockout of PI16 in Treg cells on inflammatory symptoms, histopathological changes and immune cell expression in mice with enteritis and autoimmune arthritis were observed by constructing the model of antigen-induced arthritis (AIA) and colitis induced by dextran sulfate sodium salt (DSS).

**Results:**

We identified peptidase inhibitor 16 (PI16) as a negative regulator of Treg cells. Our findings demonstrate that conditional knock-out of PI16 in Tregs significantly enhances their differentiation and suppressive functions. The conditional knockout of the PI16 gene resulted in a significantly higher abundance of Foxp3 expression (35.12 ± 5.71% vs. 20.00 ± 1.61%, *p* = 0.034) in iTreg cells induced in vitro compared to wild-type mice. Mice with Treg cell-specific PI16 ablation are protected from autoimmune arthritis (AIA) and dextran sulfate sodium (DSS)-induced colitis development. The AIA model of PI16^CKO^ is characterized by the reduction of joint structure and the attenuation of synovial inflammation and in DSS-induced colitis model, conditional knockout of the PI16 reduce intestinal structural damage. Additionally, we found that the deletion of the PI16 gene in Treg can increase the proportion of Treg (1.46 ± 0.14% vs. 0.64 ± 0.07%, *p* < 0.0001) and decrease the proportion of Th17 (1.00 ± 0.12% vs. 3.84 ± 0.64%, *p* = 0.001). This change will enhance the shift of Th17/Treg toward Treg cells in AIA arthritis model (0.71 ± 0.06% vs. 8.07 ± 1.98%, *p* = 0.003). In DSS-induced colitis model of PI16^CKO^, the proportion of Treg in spleen was significantly increased (1.40 ± 0.15% vs. 0.50 ± 0.11%, *p* = 0.003), Th17 (2.18 ± 0.55% vs. 6.42 ± 1.47%, *p* = 0.017), Th1 (3.42 ± 0.19% vs. 6.59 ± 1.28%, *p* = 0.028) and Th2 (1.52 ± 0.27% vs. 2.76 ± 0.38%, *p* = 0.018) in spleen was significantly decreased and the Th17/Treg balance swift toward Treg cells (1.44 ± 0.50% vs. 24.09 ± 7.18%, *p* = 0.012).

**Conclusion:**

PI16 plays an essential role in inhibiting Treg cell differentiation and function. Conditional knock out PI16 gene in Treg can promote the Treg/Th17 balance towards Treg dominance, thereby alleviating the condition. Targeting PI16 may facilitate Treg cell-based therapies for preventing autoimmune diseases and inflammatory diseases. The research provides us with novel insights and future research avenues for the treatment of autoimmune diseases, particularly arthritis and colitis.

**Supplementary Information:**

The online version contains supplementary material available at 10.1186/s12967-024-05082-1.

## Introduction

Regulatory T cells (Tregs) play a critical role in maintaining immune homeostasis and preventing autoimmunity and inflammation [1, 2]. Tregs can be either derived from the thymus (nTreg) or induced from conventional T (Tcon) cells in the periphery (iTreg) [3]. Abnormalities in the quantity or function of Tregs have been implicated in the development of multiple autoimmune and inflammatory diseases, such as type 1 diabetes [4], systemic lupus erythematosus [5], and rheumatoid arthritis [6].

Tregs employ various mechanisms to exert their suppressive function, such as the secretion of suppressive cytokines [7], cytotoxicity, metabolic disruption, and modulation of dendritic cell function [8, 9]. Forkhead box P3 (Foxp3) is the master regulator of Tregs differentiation and function [10, 11]. Recent studies suggested that a proportion of Treg cells can lose Foxp3 expression and become unstable under inflammation conditions [12]. These CD4^+^CD25^low^Foxp3^low^ Tregs are termed "ex-Tregs" or "exFoxp3" cells, which have been identified in mouse models of diabetes [13], multiple sclerosis (EAE) [9] and rheumatoid arthritis (RA) [14]. These ex-Tregs with low or even lack Foxp3 expression can be converted into effector T cells, acquiring Th1-like [15], Th2-like [16], or Th17-like [17, 18] properties.

Foxp3 expression is tightly regulated at epigenetic, transcriptional, and posttranslational levels. It has been suggested that multiple factors, including inflammatory factors, cytokines, and metabolic mediators [19], play a critical role in regulating Foxp3 expression. For example, the deubiquitinating enzyme USP7 could be downregulated under inflammatory conditions and then facilitate the degradation of Foxp3 Treg cells [20]. As a transcription factor, the suppressor of cytokine signaling 1 (SOCS1) is essential for Tregs functions by preventing loss of Foxp3 expression and IFN-γ and IL-17A production [21]. HIF-1α could be induced by IL-6 and the TCR [22] and is critical to the function and survival of Tregs in inflamed tissues. Under hypoxic situations, HIF-1α inhibits Treg suppression function by acting as a metabolic switch for Tregs between glycolytic-driven migration and oxidative phosphorylation-driven immunosuppression [23].

Peptidase inhibitor 16 (PI16, also known as CD364, PSPBP or CRISP-1) is a cysteine-rich secretory protein (CAP) family member. It exhibits homology with other mammalian species, such as rats and humans. The identification of pi16 in recurrent prostate cancer has established its significance as a prognostic indicator, exhibiting a positive correlation with relapse-free survival [24, 25]. Subsequently, PI16 was highly upregulated in cardiac disease and can inhibit the growth of cardiomyocytes [26] or attenuated left ventricular injury and remodeling after myocardial infarction [27, 28]. In recent research, the function of PI16 in different organs and tissues is gradually being elucidated. The subset of reticular cells expressing peptidase inhibitor 16 (PI16^+^ RC) in adult tonsils demonstrated the most pronounced structural remodeling associated with inflammation [29]. When deletion PI16 gene of mice can resist persistent inflammatory pain [30]. PI16 also plays a tumor suppressor role in BLCA by inhibiting the activation of the NF-КB pathway [31]. In our previous investigations, we have detected the presence of PI16 in the peripheral blood mononuclear cells (PBMC), serum, and synovial tissue of individuals afflicted with rheumatoid arthritis (RA) or collagen-induced arthritis (CIA) in murine models. This protein exhibits increased expression in the spleen and synovial tissues of mice, contributing to the pathological processes associated with rheumatoid arthritis/collagen-induced arthritis (RA/CIA). Following this, we generated PI16^Tg^ mice and subsequently found that PI16 exacerbates disease severity and induces bone destruction in a murine model of antigen-induced arthritis (AIA). Furthermore, the application of flow cytometry analysis revealed a decrease in the percentages of regulatory T cells (Tregs) following the upregulation of PI16 expression. After conducting a comprehensive analysis of the impact of PI16 on regulatory T cells (Tregs), it was determined that this protein has the ability to modulate the expression of Foxp3 by specifically interacting with histones located in the promoter region of Foxp3. One potential regulatory mechanism involves the modulation of K48 ubiquitin degradation, which is facilitated by Bmi-1 [32].

To further elucidate the role of PI16 on Treg and inflammation, we constructed mice with depletion of PI16 in Treg cells (PI16^CKO^). In the current study, we have found conditional knock-out PI16 in Tregs can significantly promote the differentiation and functions of Tregs and suppress autoimmune arthritis (AIA) and dextran sulfate sodium (DSS)-induced colitis development. These findings have revealed a previously unreported function of PI16, an essential negative regulator of the Tregs.

## Materials and methods

### Mice

First, cross mice carrying LoxP-flanked PI16 alleles (PI16^fl/fl^) with Foxp3 Cre (Foxp3^Cre^) mice in which Cre fusion protein is knocked into the Foxp3 gene, to generate mice with Treg-specific deletion of PI16 (PI16^fl/fl^Foxp3^Cre^, PI16^CKO^). PI16^CKO^ mice were constructed in GemPharmatech Co., Ltd (Nanjing, China). The genotypes of transgenic mice were also determined (Additional file 1: Fig. S1). All the experiments were performed with male mice (6–8 weeks old). The Animal Welfare Ethics Committee of Nanjing Medical University reviewed all experiments. All animals are raised in the SPF animal room of the Experimental Animal Center of Nanjing Medical University. All experiments were conducted according to the guidelines of ethical regulations for institutional animal care (Ethical number: IACUC 1709032-2).

### Cell preparation and flow cytometry

The lymph nodes and spleens were surgically excised and subsequently homogenized in a mouse lymphocyte separation solution to generate a cell suspension. This suspension was then filtered through a 40 μm Falcon cell strainer. The cell suspension is carefully added above the liquid level of the Lymphocyte Separation Medium (Dakewe, #7211012) to achieve stratification before centrifuging at 800×*g* for 30 min. Subsequently, the intermediate layer containing lymphocytes was carefully collected, washed, and subjected to further centrifugation following the manufacturer's instructions. Similarly, single-cell suspensions of lymph nodes were obtained [33, 34]. Subsequently stained with anti-mouse antibodies (Conjugated fluorescence, manufacturer, and catalog number shown in brackets) targeting. CD4 (FITC, eBioscience, #11-0041-85), CD4 (PC-5.5, Biolegend, #100434), CD25 (APC, eBioscience, #17-0251-82), Foxp3 (PE, eBioscience, #12-5773-82), IL-17A (PE, eBioscience, #12-7177-81), IL-4 (PE, eBisoscience, #12-7041-82), IFN-γ (PE, eBioscience, #12-7311-82), Helios (FITC, Biolegend, #137214), PD-1 (PC5.5, Biolegend, #135208), CD44 (PE, eBioscience, #12-0441-81), CD62L (FITC, Biolegend, #104405). CD4^+^CD25^+^ Treg cells and CD4^+ ^CD25^−^ Teff cells were isolated using the Mouse CD4^+^CD25^+^ Regulatory T Cell Isolation Kit II (EasySep™, #18783). Flow cytometry was utilized to measure cell surface markers, as previously noted [35]. The Foxp3 Transcription Factor Staining Buffer Set (eBioscience, #00-5523-00) was utilized for intracellular Foxp3 and Helios staining. To perform intracellular cytokine staining, including IL-17A, IL-4, and IFN-γ, cells were stimulated with phorbol myristate acetate (25 ng/mL) and ionomycin (1 μg/mL) in a cell incubator for 1 h, followed by the addition of brefeldin A (10 μg/mL) and a four-hour incubation. After thorough washing, cells were stained for surface antigens, fixed at room temperature for 25 min, permeabilized, and stained with monoclonal antibodies diluted in PBS against cytokines. Flow cytometric analysis was conducted using Fortessa with Diva software and analyzed with FlowJo. And FACS gating strategies were presented in supplementary data (Additional file 1: Fig. S2).

### In vitro Treg-cell differentiation

Mouse naive CD4^+^ T cells were isolated from single-cell suspensions of spleens obtained from PI16^CKO^ or PI16^fl/fl^ mice using the Mouse Naive CD4^+^ T Cell Isolation Kit. The isolated cells were cultured in a complete AIMV medium (Life Technologies) and stimulated with plate-coated anti-CD3 (10 μg/mL) and anti-CD28 (3 μg/mL) antibodies (Th0 conditions), or in the presence of mouse IL-2 (20 ng/mL) and human TGF-β (5 ng/mL) (Treg conditions) [36, 37]. If necessary, 0.5 mL of fresh medium was added 48 h later. After 72 h, the cells were collected and subjected to fluorescence-activated cell sorting (FACS) analysis.

### In vitro Treg suppression assay

First, we collect sorted Teff cells suspension and dilute to CFSE dye (Biolegend, #423801) with PBS to make a 5 mM solution. The Teff cells are labeled in accordance with the instructions provided by the manufacturer. CFSE (Biolegend, #423801)-labeled CD4^+^CD25^−^ Teff cells (4 × 10^5^ cells/mL, 50 μL) alone or cocultured with Treg cells (50 μL) at different ratios (0:1, 1:1,1:2, 1:4, 1:8, Treg: Teff) in the presence of 2 × 10^6^ cells/mL (50 μL) APCs and medium containing 4 μg/mL anti-CD3 antibody (50 μL) in a 96-well U-bottom plate. CFSE dilution was detected three days later, indicating the Treg-cell suppression ability [38, 39].

### Antigen-induced arthritis (AIA)

AIA mice were induced, as previously reported [40]. Briefly, methylated bovine serum albumin (mBSA, Sigma-Aldrich) was dissolved in ddH_2_O and completed Freund's adjuvant (CFA, Sigma-Aldrich). The emulsified mixture was intradermally administered to PI16^CKO^ mice on days 0 and 14 at the base of the tail (200 μL/animal). On day 21, a booster dose of mBSA in CFA emulsion was given. Measure the knee joint diameter of mice every day since day 21. At the end of the experiment, mouse knees were removed for HE at indicated days, and spleens were used to analyze by flow cytometry. (PI16^CKO^ AIA = 10, PI16^fl/fl^ AIA = 10, PI16^CKO^ NC = 3, PI16^fl/fl^ NC = 3).

### Dextran sulfate sodium salt (DSS) induced colitis

Dss-induced model mice were induced as previously reported [41]. Briefly, DSS was dissolved with bacteria-free ddH_2_O at 2.5%. The mice were divided into PI16^fl/fl^ NC (n = 7), PI16^CKO^ NC (n = 7), PI16^fl/fl^ DSS (n = 8), and PI16^CKO^ DSS (n = 9) and measured weight as baseline weight. Subsequently, the drinking water of mice in the PI16^fl/fl^ DSS and PI16^CKO^ DSS groups was replaced with 2.5% DSS water solution, and the other two groups still had normal drinking water. Let mice drink freely and add water solution in time. Evaluate the situation and weight of mice every day. The water was replaced with normal drinking water on the seventh day, the mouse distal colon was removed for HE, and spleens were used to analyze by flow cytometry on the tenth day.

### Histopathology

Joints and distal colon were removed from the autoimmune disease model or health control mice. Before tissue histology, samples were formalin-fixed, paraffin-embedded, and stained with hematoxylin and eosin. Photomicrographs were taken at 40× and 100× magnifications. Two pathologists in double-blind condition followed the standard of evaluation colitis histopathology score [42] for scoring.

### Statistics

Measured data were described as Mean ± SEM. The assumption of normal distribution was initially evaluated for all presented data. For datasets that adhere to the normal distribution, P values were calculated using two-tailed unpaired Student's t-tests. For more than two group comparisons were calculated using 2-way analysis of variance was performed with Prism (GraphPad 9.5.1) to calculate the statistical significance of the difference in mean values and P values. A P value of < 0.05 was considered statistically significant. *: p < 0.05, **: p < 0.01, ***: p < 0.001, ****: p < *0.0001.*

## Results

### PI16-deficiency in Treg promotes the differentiation and proliferation of Treg

**Fig. 1:**
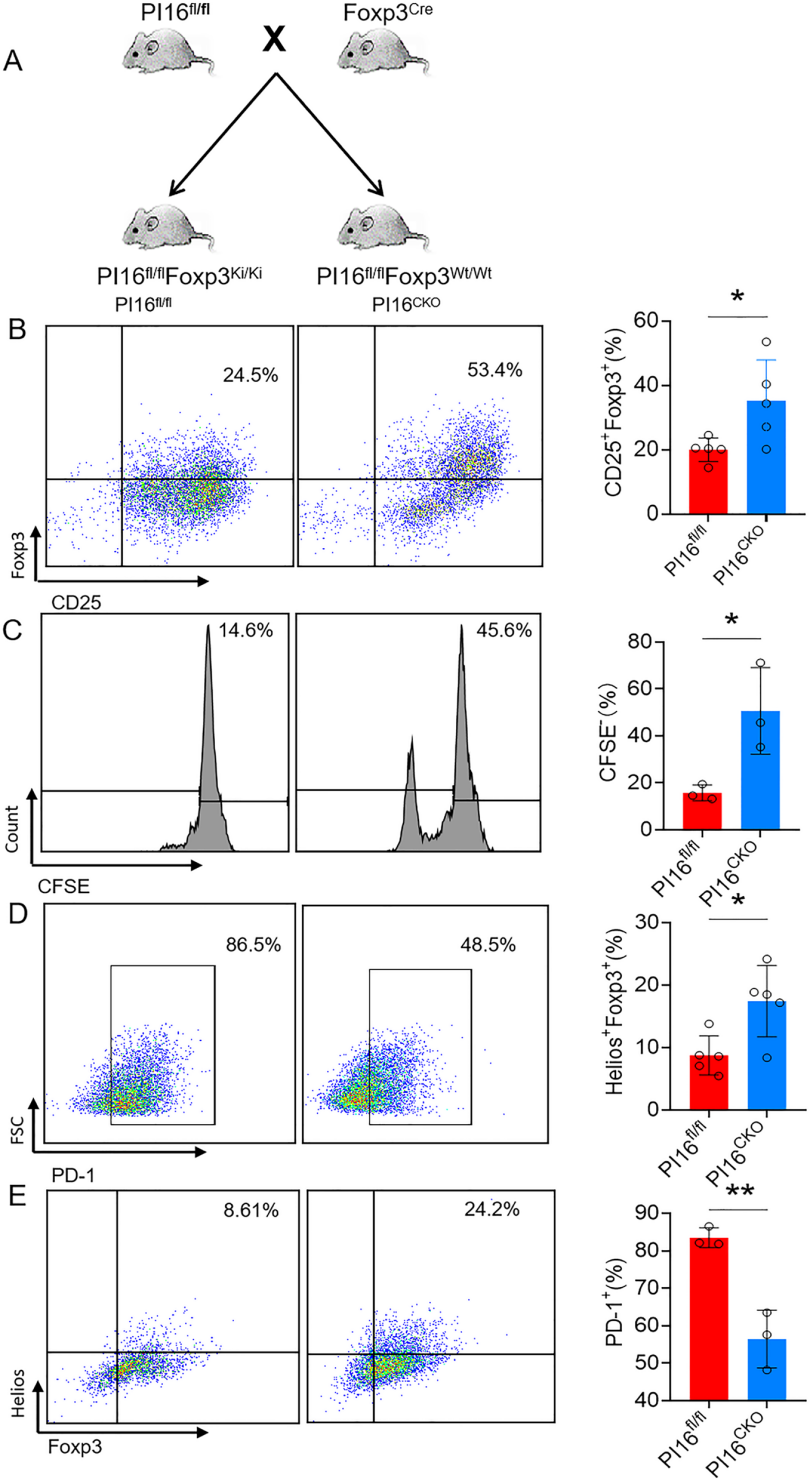
PI16-deficiency in Treg promotes the differentiation and proliferation of Tregs. **A** Diagram for the construction of PI16 conditional knockout mice; **B** expression level of Foxp3 (n = 5, 35.12 ± 5.71% vs. 20.00 ± 1.61%, *p* = 0.034) in vitro Treg induction assay; **C** the proliferation level of iTreg (n = 3, 50.11 ± 12.33% vs. 11.47 ± 2.41%, *p* = 0.037); **D**, **E** PD-1 (**D**, n = 3, 50.11 ± 12.33% vs. 11.47 ± 2.41%, *p* = 0.005) and Helios expression (**E**, n = 5,17.4 ± 2.56% vs. 8.75 ± 1.40%, *p* = 0.018) in vitro Treg induction assay. *p < 0.05, **p < *0.01*

To gain insight into PI16 functions in Treg cells, we generated Treg-conditional PI16 knock-out mice by crossing the PI16-floxed mice with Foxp3-Cre mice (PI16^CKO^) (Fig. 1A). PI16-deficiency in Treg did not affect the number of lymphocytes in the spleen and thymus. The ablation of PI16 also did not change the development of Th17, Th1, and Th2 in Tregs (data not shown). Our analysis then focused on the impact of PI16 on Foxp3 expression and Treg differentiation. Following the stimulation of naive CD4^+^ T cells under Treg skewing conditions for four days, a significant increase in the proportion of CD4^+^ CD25^+^ Foxp3^+^ Treg was observed in PI16^CKO^ mice (Fig. 1B). Similarly, in vitro Treg-cells differentiation assay showed that CFSE^+^ Treg cells were markedly increased in PI16^CKO^ mice as compared with those in PI16^fl/fl^ mice (Fig. 1C).

Helios is a marker for T cell activation and proliferation and is essential for the inhibitory activity of Treg [43]. PD-1 expression on the Treg surface was negatively correlated with the Treg function [44]. We further analyzed the role of PI16 on these surface markers related to Tregs' function. We found that the absence of PI16 in Tregs significantly increased Helios expression (Fig. 1D). In contrast, the expression of PD-1 was reduced in PI16^CKO^ Tregs (Fig. 1E). These data suggested that the lack of PI16 in Tregs could enhance the differentiation and proliferation of Treg.

### PI16-deficiency in Treg enhances the suppressive functions of Tregs

**Fig. 2:**
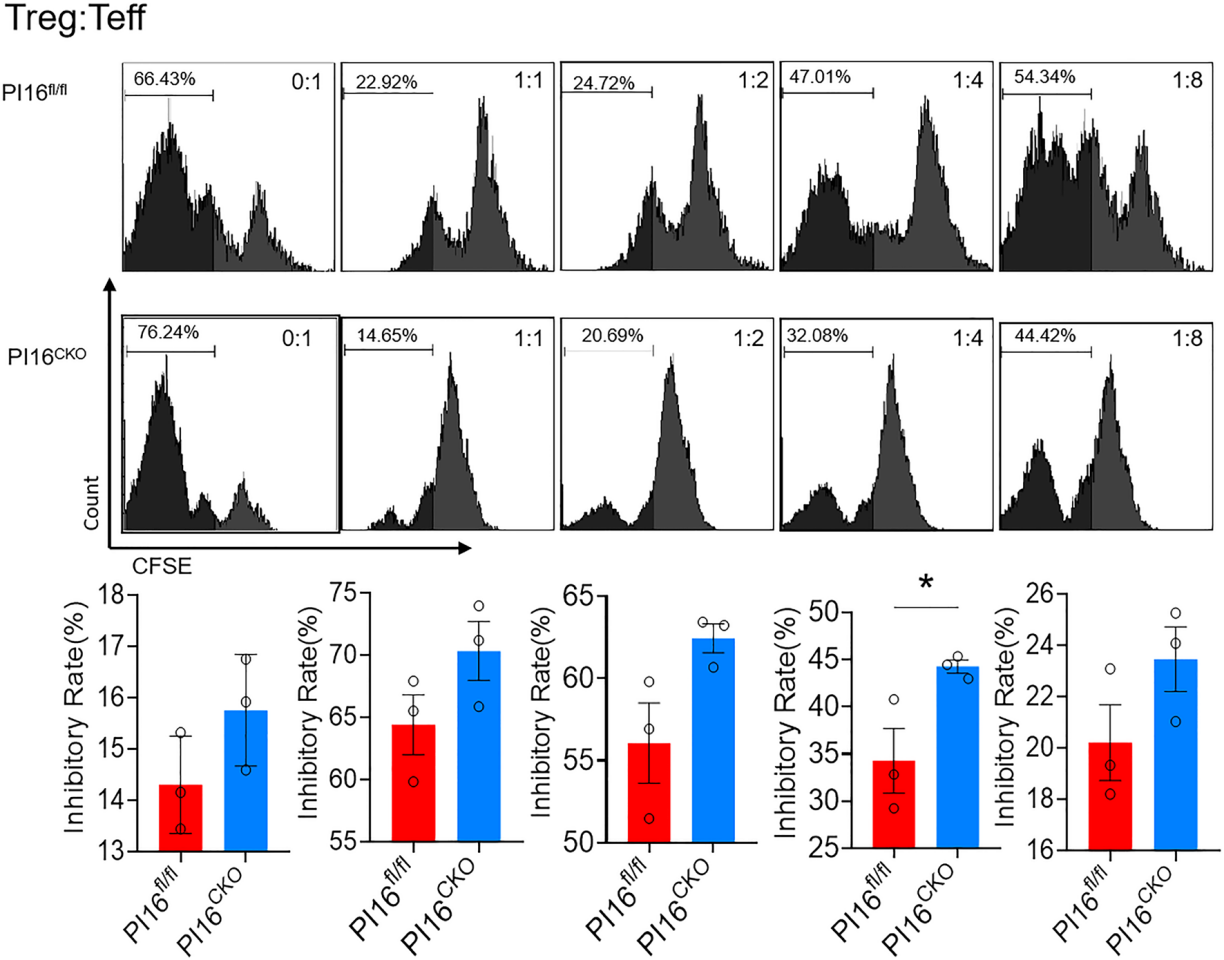
PI16-deficiency in Treg enhances the suppressive functions of Tregs. Mitomycin-treated CD4^+^ cells (APC) and CD4^+^CD25^−^ effective T cells were cocultured with Tregs that sorted from PI16^CKO^ or PI16^fl/fl^ mice (n = 3) at different Treg/Teff ratios (0:1, 1:1, 1:2, 1:4, 1:8). ns: *p* > 0.05, *p < 0.05

Given the above result, which shows that the absence of PI16 in Tregs leads to increase cell differentiation and proliferation in vitro, we next examine whether PI16 affects the suppression function of Tregs. We conducted in vitro suppression assays by coculturing mitomycin-treated CD4^+^ cells (APC) and CD4^+^CD25^−^ effective T cells with Tregs sorted from PI16^CKO^ or PI16^fl/fl^ mice at different Treg/Teff ratios. And we evaluated the purity of Teff cells before co-incubation (Additional file 1: Fig. S3). The results showed that compared with PI16^fl/fl^ mice, PI16-deficient Tregs show an increased inhibitory effect on Teff proliferation at different Treg/Teff ratios. At a ratio of 1:4, PI16^CKO^ Tregs could significantly suppress the proliferation of Teff cells (Fig. 2).

### Mice with Treg cell-specific PI16 ablation are protected from autoimmune arthritis

**Fig. 3:**
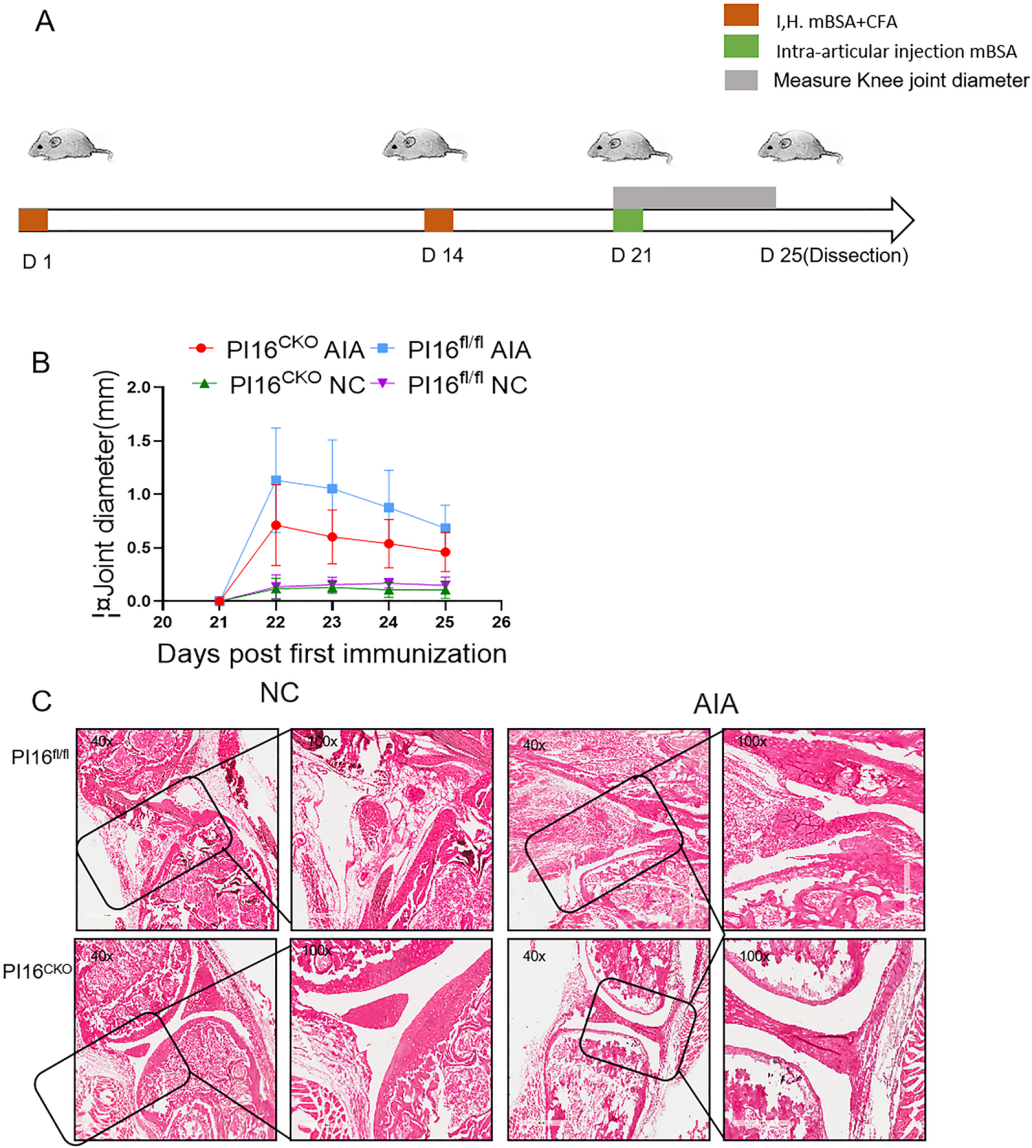
Mice with Treg cell-specific PI16 ablation are protected from autoimmune arthritis. **A** Diagram for the induction of AIA model; **B** severity of arthritis in AIA mice (n = 6) was evaluated by determining the diameter of the knee joint from the 21st day after the third immunization (n = 5); **C** representative images showed the H&E analysis for histopathological changes PI16^CKO^ or PI16^fl/fl^ mice (n = 4) (The magnification of the first and third line is 40×; the magnification of the second and fourth line is 100×)

We previously showed that PI16 was highly expressed in RA patients' serum, synovial fluid, and synovial tissue compared to these in osteoarthritis patients and health controls [32]. We suspected PI16 is involved in the inflammatory process of RA. We used the AIA model to explore the effect of PI16 on arthritis development. The modeling process of the AIA model is shown in the figure (Fig. 3A). It was found that the knee diameter of PI16^CKO^AIA model mice at 25th days had a decreasing trend compared with PI16^fl/fl^ mice (Fig. 3B). Pathological staining of mouse knee joint tissue showed a noticeable reduction in synovium thickening and inflammatory cells infiltration in PI16^CKO^ mice, as compared with those in PI16^fl/fl^ mice (Fig. 3C).

**Fig. 4:**
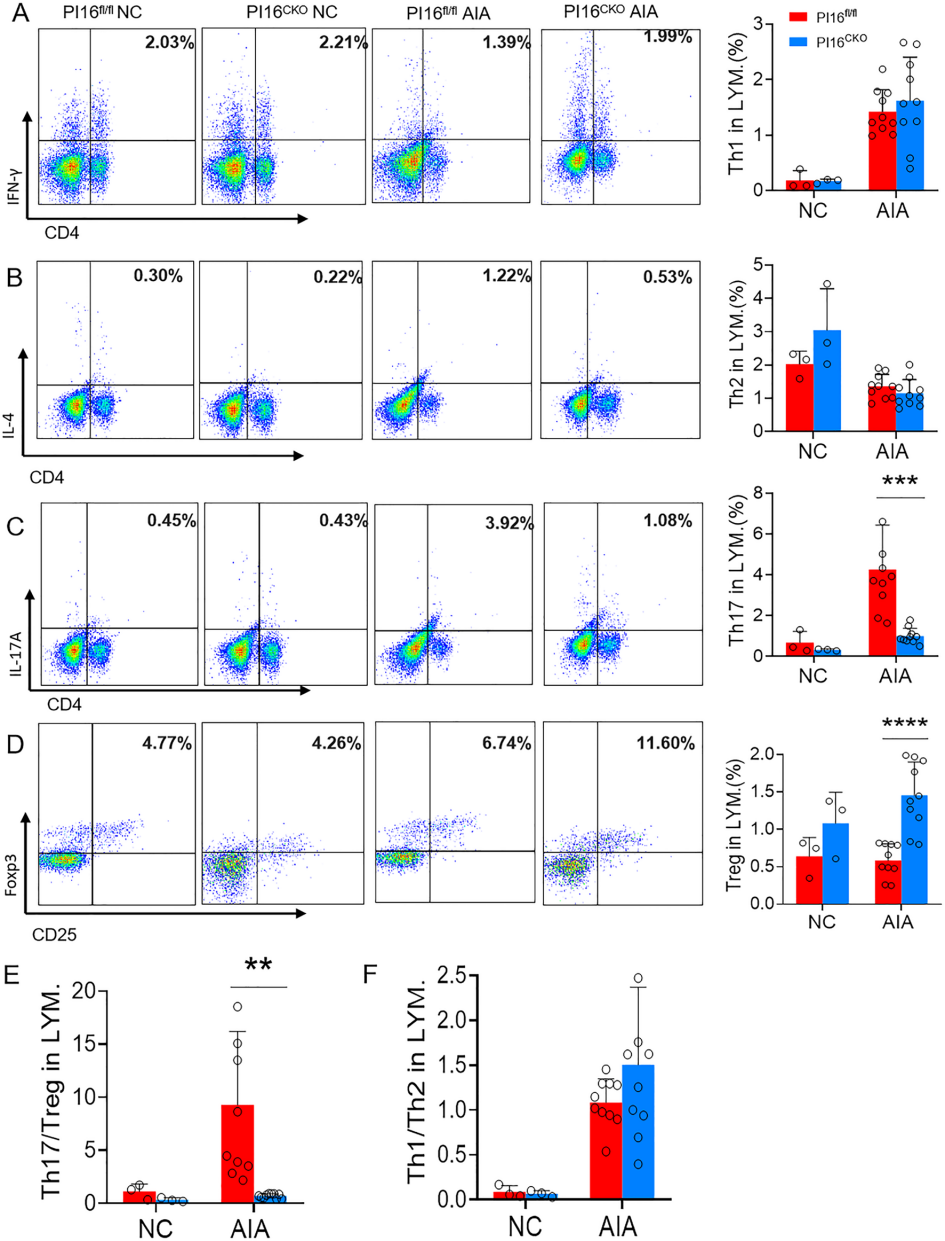
The proportion of T cell subsets in PI16^CKO^ and PI16^fl/fl^ AIA mice. The expression level of Th1 (**A**, 1.62 ± 0.25% vs. 1.61 ± 0.19%, *p* = 0.968), Th2 (**B**, 1.15 ± 0.13% vs. 1.22 ± 0.13%, *p* = 0.727), Th17 (**C**, 1.00 ± 0.12% vs. 3.84 ± 0.64%, *p* = 0.001), and Treg (**D**, 1.46 ± 0.14% vs. 0.64 ± 0.07%, *p* < 0.0001) in AIA model mice; **E**, **F** Th17/Treg balance (**E**, 0.71 ± 0.06% vs. 8.07 ± 1.98%, *p* = 0.003) and Th1/Th2 balance (**F**, 1.71 ± 0.43% vs. 1.51 ± 0.27%, *p* = 0.018) in AIA model mice. (PI16^CKO^ AIA = 10, PI16^fl/fl^ AIA = 10, PI16^CKO^ NC = 3, PI16^fl/fl^ NC = 3) *p < 0.05, **p < 0.01, ***p < 0.001, ****p < *0.0001*

Subsequently, we analyzed the proportion of T cell subsets between PI16^CKO^ and PI16^fl/fl^ AIA mice. Th1 (Fig. 4A) and Th2 (Fig. 4B) cell proportion in the spleen had no significant difference between PI16^CKO^ and PI16^fl/fl^ arthritis mice. However, Th17 (Fig. 4C) significantly decreased, and Treg (Fig. 4D) markedly increased in PI16^CKO^ compared with PI16^fl/fl^ arthritis mice. Accordingly, the Th17/Treg ratio was increased considerably in PI16^CKO^ mice (Fig. 4E). The balance of Th1/Th2 (Fig. 4F) in mice has no significant difference. These data have shown that deleting the PI16 gene in Treg can significantly reduce the severity and inflammation level in AIA arthritis mice by shifting Th17/Treg toward Treg cells.

### Mice with Treg cell-specific PI16 ablation are protected from DSS-induced ulcerative colitis

**Fig. 5:**
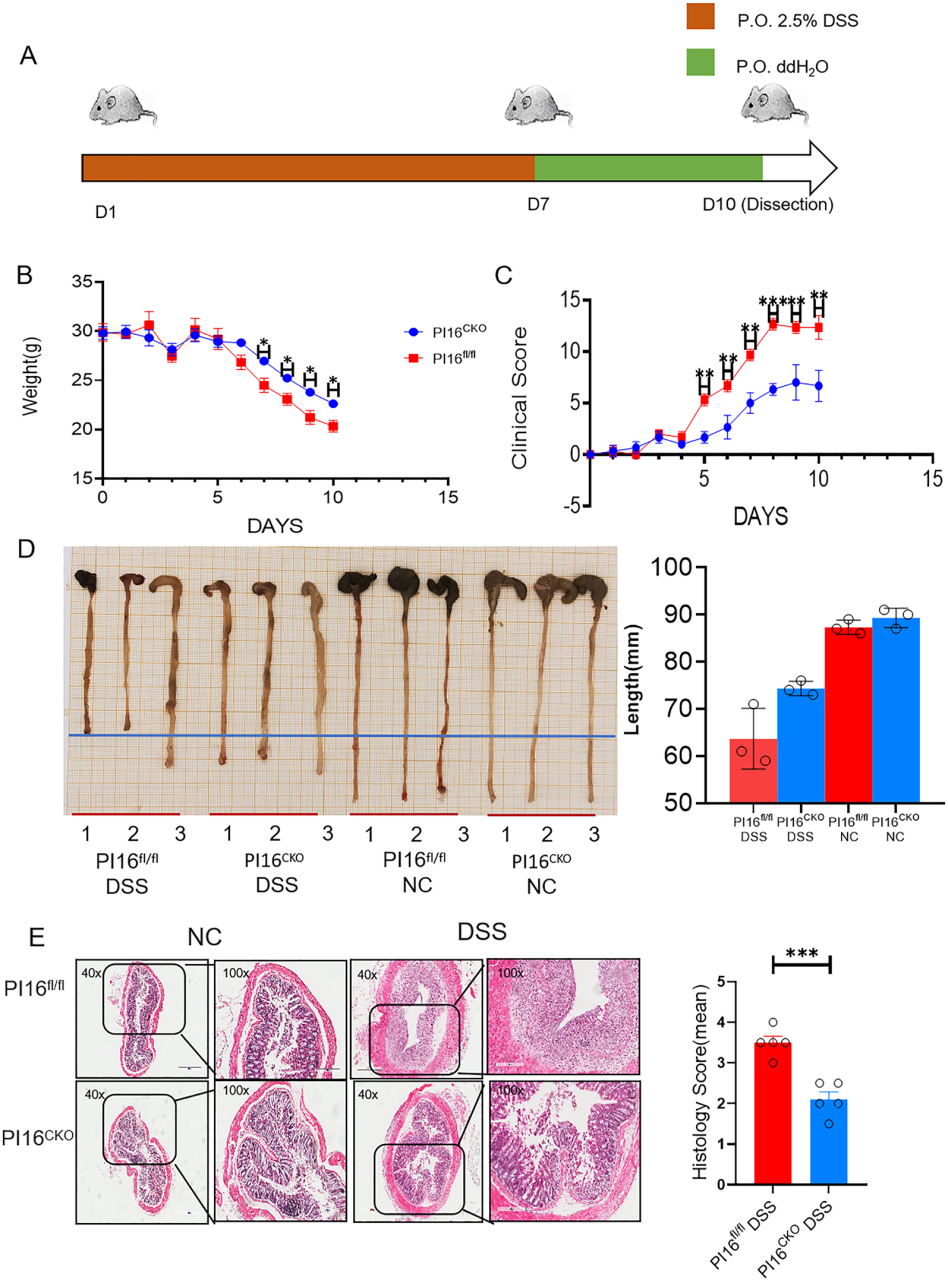
Mice with Treg cell-specific PI16 ablation are protected from DSS-induced ulcerative colitis. **A** Diagram for the induction of DSS-induced colitis model; **B** the level of body weight in PI16^CKO^ and PI16^fl/fl^ mice during the colitis model process; **C** Clinical score of DSS-induced colitis in PI16^CKO^ and PI16^fl/fl^ mice. **D** The length of the distal colon of DSS-induced colitis in PI16^CKO^ and PI16^fl/fl^ mice. Graphs show the quantitation data derived from the left figure (n = 3, 63.33 ± 3.38 vs. 73.33 ± 1.86, *p* = 0.061); **E** HE staining of distal colon harvested on day 10 of DSS treatment (The magnification of first and third line is 40×; the magnification of second and fourth line is 100×) and Histology score (n = 5, 3.50 ± 0.16 vs. 2.10 ± 0.19, *p* = 0.0007) *ns: p* > 0.05, *p < 0.05, **p < 0.01, ***p < *0.001*

Next, we confirmed the role of PI16 on inflammation response in another model of dextran sulfate sodium salt (DSS) induced ulcerative colitis model, a mouse model of inflammatory bowel disease (IBD) (Fig. 5A). Compared with PI16^fl/fl^ colitis mice, PI16^CKO^ mice had lighter weight loss degree (Fig. 5B), lower disease score (Fig. 5C), and lighter colon length shortening (Fig. 5D). These results indicated that PI16^CKO^ mice had fewer colitis symptoms than PI16^fl/fl^ mice.

Subsequently, we conducted HE staining on the end of the colon and analyzed the effect of PI16 on the degree of inflammatory infiltration and intestinal structure damage in the two groups. The end of the intestinal structure is complete, and the degree of inflammatory cell infiltration is significantly lighter (Fig. 5E) in PI16^CKO^ mice compared with PI16^fl/fl^ colitis mice.

**Fig. 6:**
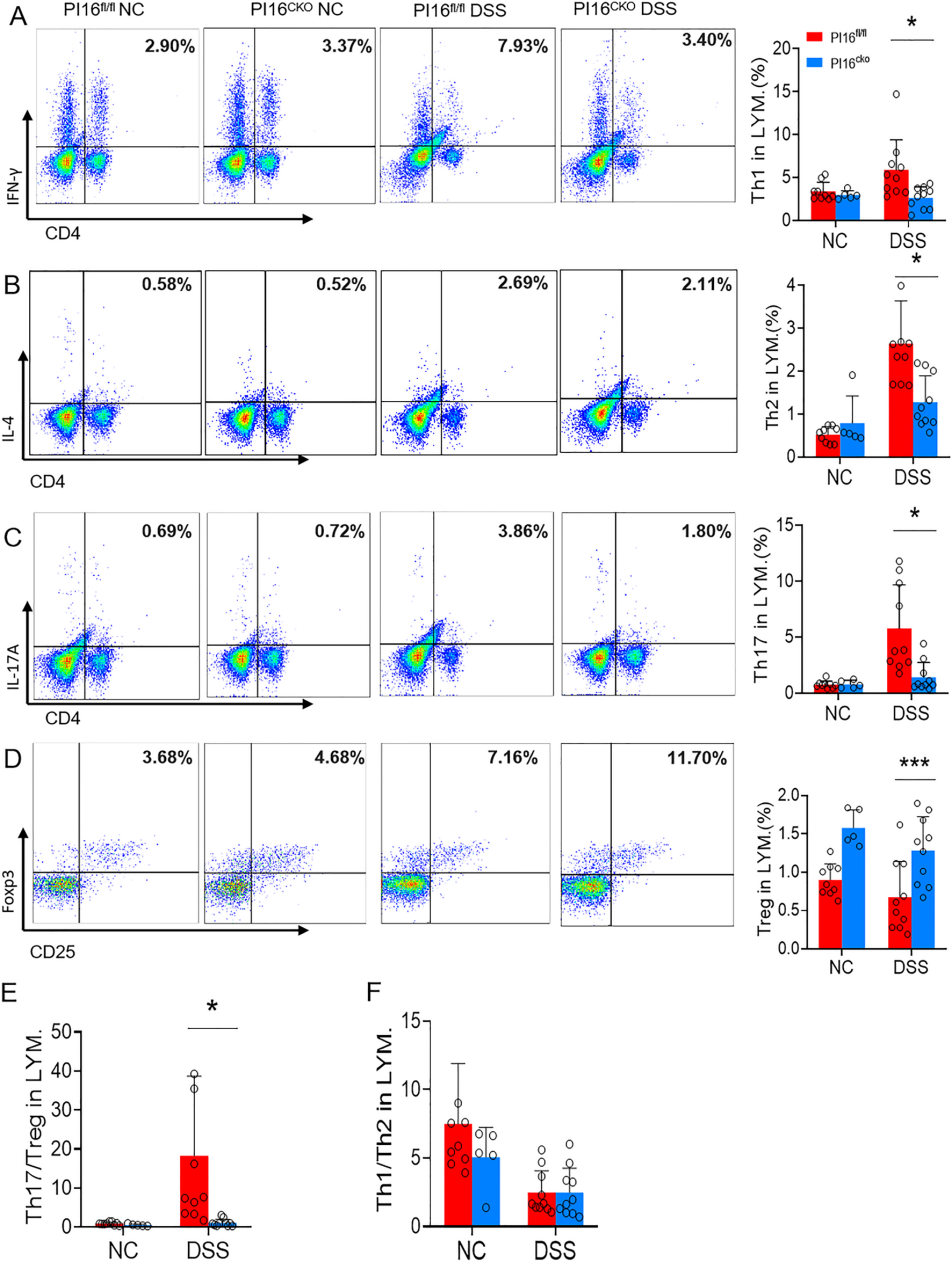
The proportion of T cell subsets in PI16^CKO^ and PI16^fl/fl^ DSS-induced colitis model. **A**–**D** The expression level of Th1 (**A**, 3.42 ± 0.19% vs. 6.59 ± 1.28%, *p* = 0.028), Th2 (**B**, 1.52 ± 0.27% vs. 2.76 ± 0.38%, *p* = 0.018), Th17 (**C**, 2.18 ± 0.55% vs. 6.42 ± 1.47%, *p* = 0.017), and Treg (**D**, 1.40 ± 0.15% vs. 0.50 ± 0.11%, *p* = 0.003) in DSS-induced colitis model mice; **E**, **F** Th17/Treg balance (**E**, 1.44 ± 0.50% vs. 24.09 ± 7.18%, *p* = 0.012) and Th1/Th2 balance (**E**, *p* > 0.05) in DSS-induced colitis model mice. (PI16^CKO^ DSS = 8, PI16^fl/fl^ DSS = 8, PI16^CKO^ NC = 7, PI16^fl/fl^ NC = 7) *p < 0.05, **p < 0.01, ***p < *0.001*

The proportion in Treg, Th1, Th2, and Th17 was significantly decreased, but the Treg proportion was markedly increased (Fig. 6A–D) in the spleen of PI16^CKO^ colitis mice than those in PI16^fl/fl^ colitis mice. Treg/Th17 balance inclines toward the Treg direction (Fig. 6E), and Th1/Th2 balance has no significant difference (Fig. 6F). These results suggested that conditional knock-out PI16 gene in Treg could protect mice from DSS-induced ulcerative colitis by promoting the shift of Th17 toward Treg cells.

## Discussion

The stable inhibitory functions of Treg are essential for controlling inflammation and preventing autoimmune disorders, infectious diseases, and cancers A reduction in the expression of Foxp3 or the frequency of Tregs has been observed in various diseases, including RA, type 1 diabetes mellitus, and multiple sclerosis [45–47]. However, the precise mechanism underlying this phenomenon remains unclear. In the current study, we have found that the conditional knock-out of PI16 in Tregs can faciliate the differentiation and proliferation of Tregs and augment their suppressive functions. Furthermore, the present study reveals that mice with Treg cell-specific PI16 ablation exhibit protection against autoimmune arthritis and DSS-induced colitis through the modulation of Th17/Treg balance towards Treg cells. These findings provide evidence that PI16 functions as a negative regulator of Treg cell differentiation and function, and targeting PI16 may offer potential benefits for Treg cell-based therapies in the context of autoimmune and inflammatory diseases.

Although the role of PI16 on immune cells remains largely unexplored, the current investigation focuses on Tregs due to previous indications of a relationship between PI16 and these cells.The presence of PI16 in AIA models of arthritis plays a significant role in both joint inflammation and damage. Additionally, our research conducted with PI16^Tg^ mice has suggested a correlation between elevated PI16 expression in inflammatory arthritis and decreased Foxp3 expression in Tregs, thereby potentially playing a role in the advancement of the disease. Our proposition also suggested that inflammation plays a role in the interaction and stabilization of Bmi-1 by PI16 in Treg cells, leading to the promotion of a repressive histone modification in the Foxp3 promoter and ultimately resulting in the loss of Foxp3 expression. Therefore, focusing on the PI16-Bmi-1 pathway could potentially offer a viable therapeutic strategy for alleviating inflammation in arthritis through the restoration of Treg function [32]. Our findings indicate that the absence of PI16 in Tregs results in a significant increase in Treg generation. This augmented proliferative capacity of PI16-deficient Tregs confers greater suppressive ability on the proliferation of Teff compared to Tregs from PI16^fl/fl^ mice.

Additionally, the expression of Helios in Treg from PI16^CKO^ mice was higher, but the surface labeled PD-1 was lower than that in Treg from PI16^fl/fl^ mice. Although the function of Helios in Tregs is not fully understood, Helios expression is associated with T cell activation and cellular division and is considered a marker for stable Foxp3 expression [48]. Previous studies have shown that Helios^+^ Treg has a more vital ability to inhibit inflammatory immune cells and cytokine levels than Helio- Tregs under inflammatory conditions [49, 50]. These results support the notion that PI16 might serve as a negative modulator of Tregs proliferation and function. It is evident that Helios^+^ and Helios^−^ CD4^+^ Treg lymphocytes possess significant distinctions in terms of their phenotypic, genetic, and functional characteristics [48, 51]. CD4^+^ Tregs expressing Helios demonstrate a more activated phenotype, characterized by a higher proportion of effector cells. Additionally, in vitro investigations indicate that these Tregs exhibit a stronger immunosuppressive capacity compared to CD4^+^ Tregs lacking Helios [52]. Furthermore, experiments conducted on lymphopenic animals have demonstrated that CD4^+^ Tregs expressing Helios in vivo exhibit a more stable expression of FoxP3.

The PD-1/PD-L1 pathway performs a crucial role in maintaining immunological homeostasis and protects against autoimmunity through various ways, such as promoting the development and function of Treg [53] and directly inhibiting the function of peripheral potential pathogenic effector T cells [54]. However, the PD-1^+^ iTreg of PI16^CKO^ mice was significantly reduced under induced differentiation in the current study, which seemed contrary to the previous results. However, some studies have shown that the knockdown of PD-1 on the surface of Treg can activate the Treg phenotype, inhibit teff proliferation in vitro, and alleviate the severity of diabetes in EAE model mice and NOD mice [55]. Interestingly, after systemic knock-out of the PD-1 gene, the disease of the EAE model [56, 57] and diabetes model mice [58, 59] worsened. PD-1 has diverse roles in various immune cells. The lowering of PD-1 expression abundance indeed benefits the activation of the Treg phenotype and improved immunosuppression only from the standpoint of Treg function [60–62]. We hypothesize that the inhibitory effect of PD-1 on a diverse range of autoimmune cells may contribute to its role in regulating Treg function. Knock-out of PD-1 in Tregs could potentially enhance their suppressive capacity. However, systemic PD-1 knockout alters the developmental milieu and compromises Treg functionality. Consequently, despite augmented suppression by Tregs, it fails to prevent exacerbation of autoimmune disease resulting from various T cell activations following PD-1 knockout.

Although joint swelling only exhibits a tendency (Fig. 3B), PI16^CKO^ does mitigate inflammatory outcomes. This assertion is supported by our utilization of HE staining and subsequent FACM data. Upon removal of the PI16 gene in Treg cells, there was a notable reduction in both bone destruction and synovial swelling in arthritis, accompanied by a less severe manifestation of immune disorders. Accumulating evidence suggests that Tregs and Th17 cells play crucial in the pathogenesis of rheumatoid arthritis [63–65] and IBD [66–68]. In the model of AIA and DSS-induced colitis, PI16^CKO^ mice displayed attenuated arthritis and colitis, as compared with PI16^fl/fl^ mice. Furthermore, PI16 conditional knock-out mice showed decreased Th17 cells and increased Tregs. These results suggest an essential role of PI16 in preventing inflammatory response under inflammatory conditions by regulating differentiating Treg cells and Treg/Th17 balance. Unfortunately, the specific mechanism of, as a peptide inhibitor, knocking-out PI16 how to promote Th17 to Treg remains largely unknown. We will explore this question in the future.

In conclusion, our data have disclosed a previously unreported new function of PI16 in regulating Treg differentiation and function, which will contribute to arthritis and colitis development. And PI16 may be one of the underlying mechanisms contributing to immune cell dysfunction in patients diagnosed with autoimmune diseases. Therefore, PI16 might be a potential therapeutic target for Treg cell-related inflammatory and may facilitate the development of innovative therapies targeting Tregs for Rheumatoid Arthritis (RA) and other autoimmune diseases. In the future, our focus will be directed towards elucidating the underlying mechanism by which PI16 influences the expression of Foxp3, while also exploring the potential correlation between levels of regulatory T cells (Tregs) and PI16 in patients suffering from enteritis and arthritis.

### Supplementary Information

**Additional file 1: Figure S1.** Genotype Knockout Identification of Mice. A-B PI16^Fl/Fl^ Genotype Knockout Identification. Genotype: No. 635 PI16^fl/fl^, No. 638 PI16^fl/null^; C Foxp3^Ki^ Genotype Knockout Identification. Genotype: No. 635 Foxp3^wt/wt^; No. 638 Foxp3^Cre^. Genotype: No. 635 PI16^fl/fl^Foxp3^wt/wt^(PI16^fl/fl^), No. 638 PI16^fl/null^Foxp3^Cre^( PI16^CKO^). **Figure S2.** FACM Gating Strategies. A-D. Gating Strategies of Th1 (A), Th2 (B), Th17 (C) and Treg (D). **Figure S3.** Effector T Cells Health in the Vitro Suppressive Assay.

## Data Availability

The datasets used and/or analyzed during the current study are available from the corresponding author (tw2006@njmu.edu.cn) upon reasonable request.
