# Supplementary material for: Regulatory role of PI16 in autoimmune arthritis and intestinal inflammation: implications for Treg cell differentiation and function
Source: J Transl Med. 2024 Apr 2;22:327. doi: 10.1186/s12967-024-05082-1 (PMC10985956; doi:10.1186/s12967-024-05082-1)
Supplement: Supplementary file 1 — Additional file 1: Figure S1. Genotype Knockout Identification of Mice. A-B PI16Fl/Fl Genotype Knockout Identification. Genotype: No. 635 PI16fl/fl, No. 638 PI16fl/null; C Foxp3Ki Genotype Knockout Identification. Genotype: No. 635 Foxp3wt/wt; No. 638 Foxp3Cre. Genotype: No. 635 PI16fl/flFoxp3wt/wt(PI16fl/fl), No. 638 PI16fl/nullFoxp3Cre( PI16CKO). Figure S2. FACM Gating Strategies. A-D. Gating Strategies of Th1 (A), Th2 (B), Th17 (C) and Treg (D). Figure S3. Effector T Cells Health in the Vitro Suppressive Assay. [file 12967_2024_5082_MOESM1_ESM.docx]

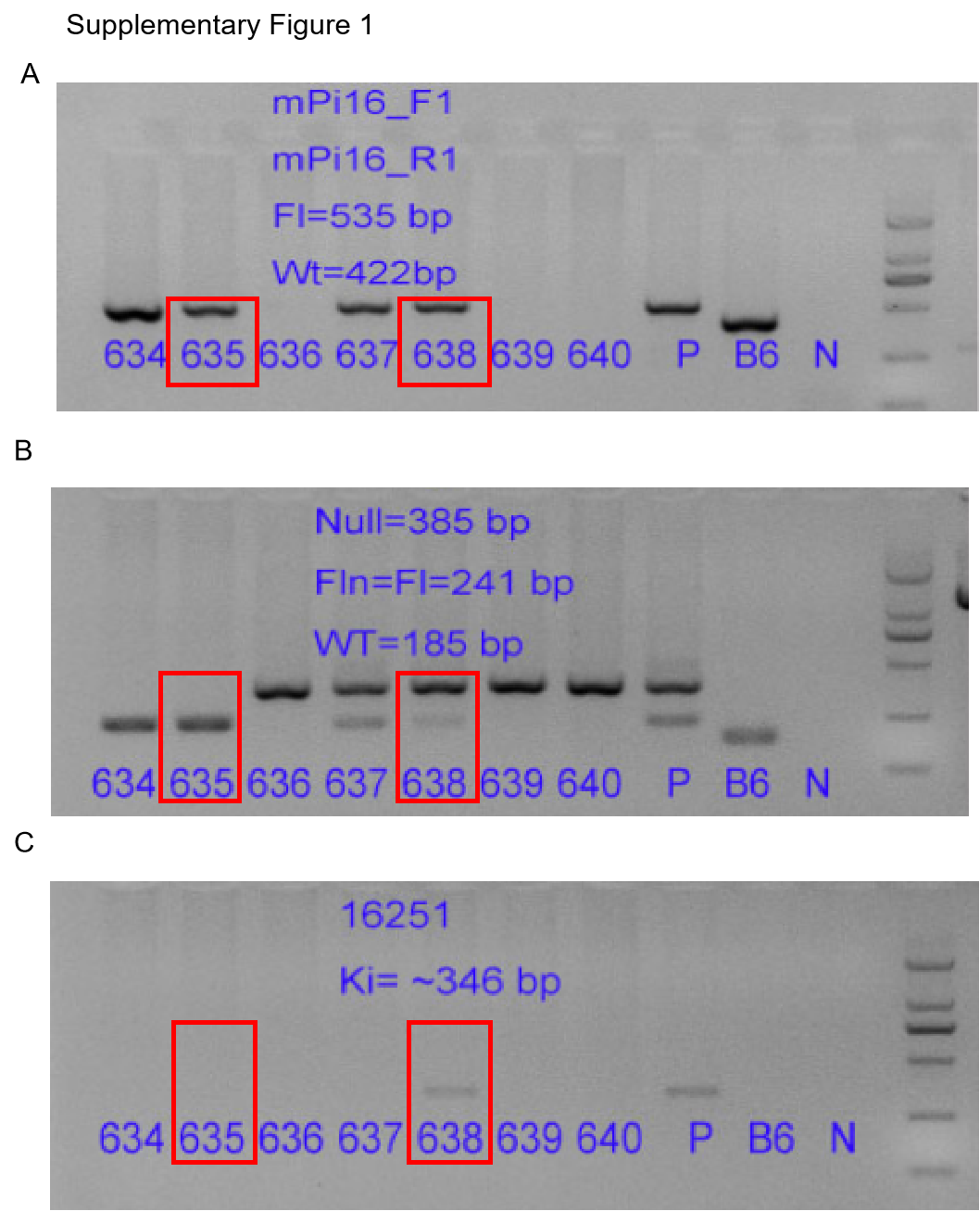


**Figure S1.** **Genotype Knockout Identification of Mice.** A-B PI16^Fl/Fl^ Genotype Knockout Identification. Genotype: No. 635 PI16^fl/fl^, No. 638 PI16^fl/null^; C Foxp3^Ki^ Genotype Knockout Identification. Genotype: No. 635 Foxp3^wt/wt^; No. 638 Foxp3^Cre^. Genotype: No. 635 PI16^fl/fl^Foxp3^wt/wt^(PI16^fl/fl^), No. 638 PI16^fl/null^Foxp3^Cre^( PI16^CKO^)


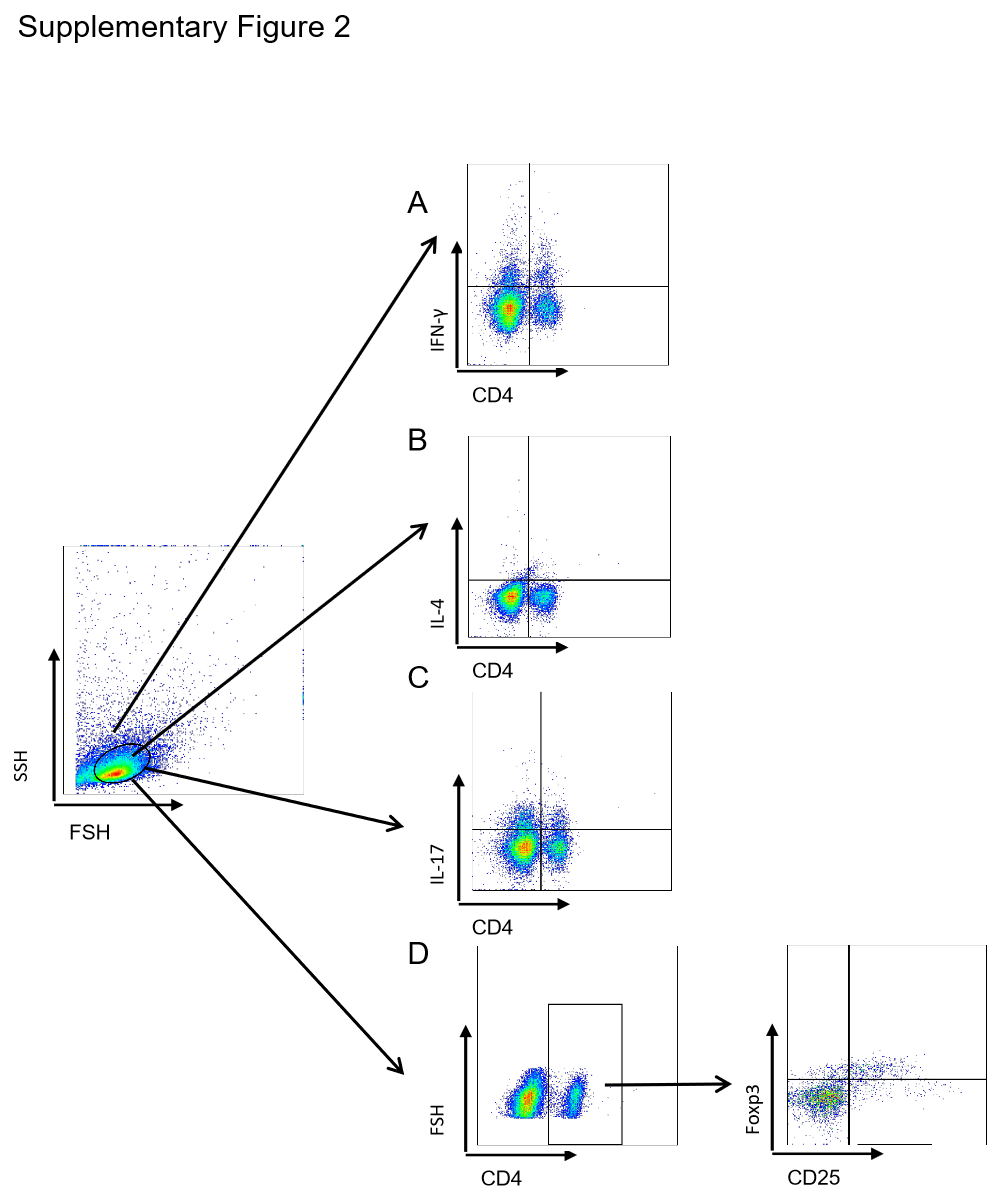


**Figure S2. FACM Gating Strategies.** A-D. Gating Strategies of Th1 ( A ), Th2 ( B ), Th17 ( C ) and Treg ( D ).

**
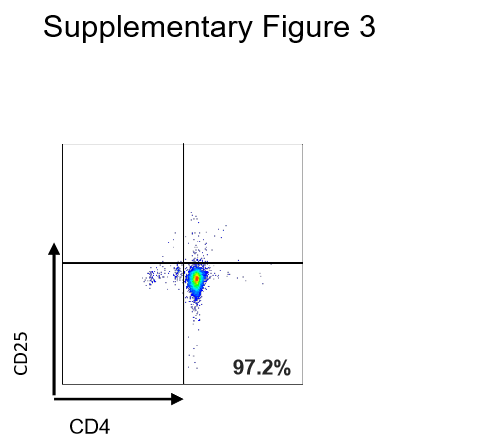
**

**Figure S3.** **Effector T Cells Health in the Vitro Suppressive Assay.**
